# Biomarker Discovery in Animal Health and Disease: The Application of Post-Genomic Technologies

**Published:** 2007-07-10

**Authors:** Rowan E. Moore, Jennifer Kirwan, Mary K. Doherty, Phillip D. Whitfield

**Affiliations:** Proteomics and Functional Genomics Research Group, Faculty of Veterinary Science, University of Liverpool, Liverpool, United Kingdom

**Keywords:** Biomarker, Transcriptomics, Proteomics, Metabolomics, Veterinary

## Abstract

**Summary:**

The causes of many important diseases in animals are complex and multifactorial, which present unique challenges. Biomarkers indicate the presence or extent of a biological process, which is directly linked to the clinical manifestations and outcome of a particular disease. Identifying biomarkers or biomarker profiles will be an important step towards disease characterization and management of disease in animals. The emergence of post-genomic technologies has led to the development of strategies aimed at identifying specific and sensitive biomarkers from the thousands of molecules present in a tissue or biological fluid. This review will summarize the current developments in biomarker discovery and will focus on the role of transcriptomics, proteomics and metabolomics in biomarker discovery for animal health and disease.

## Introduction

Biomarkers are indicators of biological processes and pathological states that can reveal a variety of health and disease traits (Biomarkers Definitions Working Group, 2001). Biomarkers are particularly relevant in medical and veterinary research where they have an important role in the characterization of human and animal diseases.

The use of animal models has been of immense value in defining and understanding human disease. Whilst the majority of these studies employ mice and rats, for some diseases, rodent models are inappropriate and large animal species may be more suitable (Hein and Greibel, 2003; Kues and Niemann, 2004; Starkey et al. 2005). In addition to providing an insight into human pathology, the outbreak of animal disease may also have a major impact on human health via exposure to hazards arising from animals, animal products and their environment (Stewart et al. 2005). These hazards may include zoonoses, vector-borne infections and other communicable diseases (Taylor et al. 2001; Kahn, 2006).

Within a veterinary setting the optimization of animal health is clearly a motivating factor. It has been estimated that almost two-thirds of U.S. households have at least one pet (http://www.appma.org/press_industrytrends.asp), whilst in the U.K. this figure is just over fifty percent (http://www.pfma.org.uk/overall/pet-ownership-trends.htm). The provision of appropriate veterinary healthcare for companion animals is therefore an important consideration. Similarly, the health and welfare of farm animals is also of major importance to agriculture. Diseases such as foot and mouth and avian influenza have significant implications for the management of livestock and poultry and can result in huge production losses and market disturbances.

The discovery of novel biomarkers for animal diseases has the potential to further enhance clinical care. Conventional analyses target a selection of biochemical or molecular biomarkers that are related to, or associated with, a specific disease state. These biomarkers play a key role in defining animal disease; however, some have poor diagnostic specificity and are not pathognomonic for the disease. Identifying novel biomarkers or biomarker profiles will be an important step towards the management of disease in animals. This review will summarize the current developments in biomarker discovery. In particular the review will focus on the role of post-genomic technologies in biomarker discovery for animal health and disease.

### Biomarker characteristics

A biomarker should possess key characteristics and qualities, which will depend upon its intended use (Aronson, 2005; LaBaer, 2005). A biomarker must be accurate, sensitive and specific. The biomarker should be altered in the relevant disease and be able to discriminate between diseased and control populations. It should also be possible to quantify the biomarker reliably and reproducibly. For diagnostic purposes biomarkers should ideally be obtained from readily accessible body fluids in animals such as blood plasma, urine, sweat and saliva or other accessible materials such as hair and feces. The identification and quantification of biomarkers also provides an opportunity to accurately assess the clinical responses to therapy and guide decisions on treatment programs. A summary of biomarker characteristics is listed in Table 1.

### Validation of biomarkers

The introduction of bias is a significant concern in the design, conduct, interpretation and reporting of any biomarker study. Bias can be unintentionally introduced at any stage of the trial, but most commonly it will be during population selection, specimen collection/storage, sample processing or sample and data analysis (Ransohoff, 2005). Therefore, once a biomarker has been identified it must be rigorously evaluated to demonstrate that it will provide an acceptable measure of a biological process or pathological state in an animal (Table 2).

At the biological level, the species, breed, sex and age of the animal should be defined as such variations may lead to marked differences in the composition of body fluids. In addition, the reproductive status of the animal, diurnal variations and its diet should be taken into account. Similarly the handling of an animal or changes to its environment can cause a stress response that may interfere with biomarker validation. It is important that a standardized protocol for sample collection, processing and storage is established in order to obtain reproducible data between laboratories, whilst the sensitivity and specificity of the assays used to measure a biomarker must also be validated.

Validation studies for biomarkers typically require large populations of animals, both healthy and diseased. This may present a major problem as case numbers can be low and the recruitment of healthy animals can be difficult to justify ethically. Control samples typically have to be collected from animals that present in the clinic with a disorder unrelated to that being studied. Excess blood or urine, which have been collected from an animal as part of routine diagnostic investigations may be of use in determining the baseline concentration of a biomarker. A U.K. DNA archive for companion animals (http://pcwww.liv.ac.uk/DNA_Archive_for_Companion_Animals/index.htm) already exists for the study of genetic disorders. This facility provides a valuable and ethical resource to assist veterinary research scientists in the study of a wide range of diseases in dogs, cats and horses.

### Biomarkers in the clinic

Ideally, a biomarker assay for an animal disease should be suitable for use in primary veterinary practice or in the field allowing clinicians to directly monitor animals for specific diseases. Urine tests lend themselves well to simple dipstick assays that combine all required reagents on a thin plastic strip. Whilst these assays deliver only crude quantification, they are rapid and may act as a starting point for future, more specialized tests. Well-established biochemical techniques, for example an enzyme-linked immunosorbent assay (ELISA) or a radioimmunoassay (RIA) are also used for characterizing markers of animal disease. Further, molecular biology techniques such as polymerase chain reaction (PCR) are becoming more routine and may enable the rapid and specific detection of animal diseases (Schmitt and Henderson, 2005).

## Post-Genomic Technologies in Animal Health and Disease

**Figure 1:**
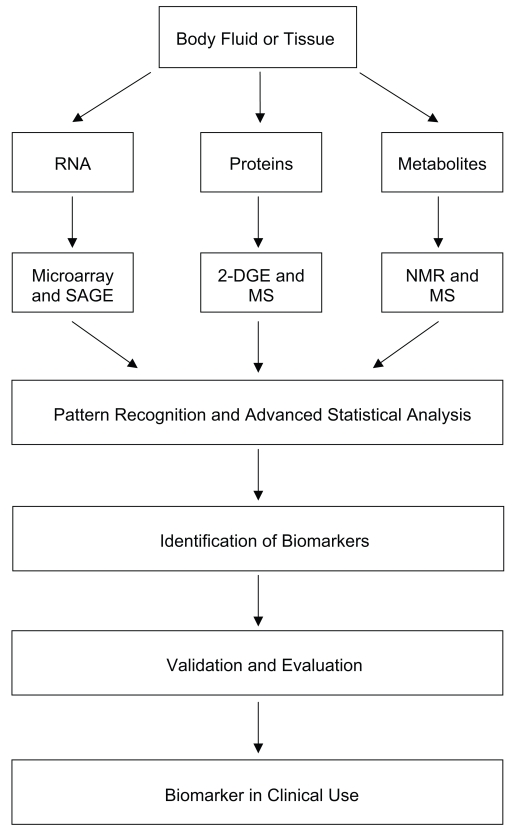
**Post-genomic approaches to biomarker discovery.** Post-genomic technologies have provided new avenues for biomarker discovery. Biological fluids and tissues hold a wealth of information at the transcript, protein and metabolite level which may be able to characterize disease states in animals. The identification of diagnostically relevant biomarkers requires rigorous validation before use in the clinic.

Global approaches that screen large numbers of molecular targets simultaneously are playing increasingly important roles as discovery tools in the basic biological and clinical sciences. In particular, the rapid advancement of the post-genomic technologies of transcriptomics, proteomics and metabolomics has led to the development of strategies aimed at identifying biomarkers from the thousands of molecules present in a tissue or biological fluid (Ilyin et al. 2004; Seo and Ginsberg, 2005) (Figure 1).

Post-genomic approaches are best addressed by integrative studies that include measurements of mRNA, proteins and low molecular weight metabolites over time and varied conditions. Bioinformatics are then used to relate these data to the genome and to the physiology or pathophysiology of the animal. Transcriptome analysis defines the population of mRNA species in a cell at a specific time and set of conditions. Proteomics addresses the technically and conceptually more challenging problem of defining changes in protein expression, dynamics and post-translational modifications, whilst metabolomics measures broad populations of low molecular weight metabolites.

Compared to their application to biomarker discovery in human medicine, reports of the use of post-genomic technologies to study animal health and disease have been limited (Witkamp, 2005). However, there is an increasing awareness and application of post-genomic strategies in veterinary research (Table 3). Whilst post-genomic technologies hold great promise, formidable challenges remain, especially in systems where analytes are uncharacterized or unknown. In this section we will summarize how transcriptomic, proteomic and metabolomic technologies have been applied to the identification of new biomarkers in animal disease states and discuss the limitations in bringing such markers into routine clinical use.

### Transcriptomics

Many traits and disease states are genetically determined, either by a single gene mutation or due to a polygenic effect. A study of a species genome and its mutations in cases of disease or breeding attributes can yield benefits. Further, the analysis of genes which display differential expression may enable the discrimination of specific disease states and provide further insight into the pathogenesis of disease states. The genomics era has heralded a wealth of information for animals of both commercial and research importance. Genome maps have been assembled for horse, cow, pig, dog, chicken and other species (Kirkness et al. 2003; Fadiel et al. 2005; Womack, 2005).

One common method of mapping and cataloguing genomic information is in the form of expressed sequence tags (ESTs), which can be used as markers and for positional mapping within a genome. A number of animal EST public databases are managed by the National Center for Biotechnology Information (NCBI) (http://www.ncbi.nlm.nih.gov/dbEST/index.html). ESTs have been useful in developing single nucleotide polymorphism (SNP) markers to allow a more refined genetic map to be produced (Snelling et al. 2005). A U.K. based consortium have also made available a transcriptome resource for chicken which has been assembled from both cDNA and EST data (Hubbard et al. 2005).

Transcriptomics monitors the expression levels of thousands of genes simultaneously at a specific time and set of conditions allowing the definition of the mRNA population. The ability to determine gene expression on a global scale has been facilitated by rapid advances in molecular technologies. The main platforms used to perform transcriptomic experiments are DNA microarrays (Schena et al. 1995) and serial analysis of gene expression (SAGE) (Velculescu, 1995).

Microarrays from both commercial and academic sources now exist for the cow, dog and chicken as well as for other animals (Cogburn et al. 2003; Suchyta et al. 2003; Holzwarth et al. 2005). There are public repository databases for farm animal genome projects such as ArkDB (Hu et al. 2001) and AgBase (McCarthy et al. 2006) and cross-species microarray chips are now becoming available for genes where there is high sequence conservation (Ji et al. 2004; Burnside et al. 2005; Grigoryev et al. 2005).

The variance between microarray data can be greatly influenced by a number of factors. These can include sample preparation, background fluorescence, together with spot-spot and array-array differences in signal intensity. Complex normalization and correction routines must be applied to the resulting data. In order for reliable comparison of transcript data obtained from microarray experiments, researchers have proposed guidelines for the reporting of such data. Minimum information about a microarray experiment (MIAME) (Brazma et al. 2001) aims to standardize annotation and exchange of microarray data. The analysis of microarray expression data also requires advanced statistical techniques for the interpretation of the data (Gracey and Cossins, 2003).

### Applications of transcriptomics to animal health and disease

Transcriptomics is a powerful approach that has many potential diagnostic applications in animal medicine (Hiendleder et al. 2005). These include scanning for gene mutations including SNPs and gene expression profiling of disease and normal conditions. Additionally, animal pathogens can be detected and genotyped using transcriptomic approaches (Feilotter, 2004). At present, it is not always practical to use these technologies directly in a clinical environment; however, transcriptomics may provide a starting point for the development of routine diagnostic tests.

#### Bovine infectious disease

Paratuberculosis or Johne’s disease is a chronic infectious disease of ruminants caused by the slow-growing intracellular bacterium *Mycobacterium avium* subspecies *Mycobacterium paratuberculosis*. A cDNA microarray approach has been used to detect changes in peripheral blood mononuclear cells in clinical and sub-clinical Johne’s disease-positive cows (Coussens et al. 2002). In an extended study (Skovgaard et al. 2006), non-stimulated leukocytes were isolated from a group of infected cows and gene expression compared to cells from control cows. Fifty-two genes were reported to be differentially expressed in leukocytes from infected cattle and quantitative-real time PCR (q-RT PCR) showed that a subset of leukocyte genes are consistently expressed at different levels depending upon infection status. Genes encoding the proteins, P-selectin, IL-1RA and CD30L were consistently expressed at a higher level within the sub-clinical group and activin RIIA was expressed at lower levels in cells from the sub-clinical group.

#### Genotyping for disease resistance in chicken

Marek’s disease is a herpes-induced T cell cancer of chickens with high economic impact on the world poultry industry. Resistance to Marek’s disease is complex and controlled by many genes, making it a difficult trait to study. Assessment of gene expression in peripheral blood lymphocytes from uninfected and Marek’s disease virus-infected inbred lines was performed using microarray technology (Liu et al. 2001). Twofold increases or decreases were searched for in autoradiographs of chicken DNA microarrays, with twenty-five genes showing increased expression and fifty-five genes showing decreased expression. Both growth hormone and lymphotactin were identified as possible candidate marker genes. Recent work has focused on the detection of Marek’s disease in chicken feather tips. The virus is carried through the bloodstream to the visceral organs, peripheral nerves and feather follicle epithelium *via* T lymphocytes. PCR methods have been developed for quantification of viral DNA from feathers (Abdul-Careem, 2006; Baigent, 2006).

### Proteomics

Proteomics is defined as the study of the protein component of a cell, tissue or organism at a given time under given conditions (Wilkins et al. 1995). A proteomic approach to biomarker discovery requires a combination of efficient and stringent separation technologies and high-resolution mass spectrometry (Aebersold and Mann, 2003; Anderson and Anderson, 2005; Duncan and Hunsucker, 2005; Rifai et al. 2006). However, these strategies bring many analytical challenges, which have yet to be fully optimized (Zolg, 2006).

The most widely used proteomics strategy for protein profiling is 2-dimensional gel electrophoresis (2-DGE) (O’Farrell, 1975; Gorg et al. 1998). 2-DGE can be limited in terms of its reproducibility and other methods such as difference gel electrophoresis (DIGE) may be employed to reduce the effects of gel-to-gel variation (Unlu et al. 1997). Proteins separated by 2-DGE are commonly identified using peptide mass fingerprinting (PMF) however, a potential difficulty with this approach is the lack of complete and annotated genome sequences of particular species. Whilst it is possible to identify proteins with high sequence conservation by PMF and cross-species matching (Liska and Shevchenko, 2003), a single amino acid change in a protein can result in dramatically different peptide mass fingerprint. The accurate identification of proteins from animals often requires *de novo* sequencing of peptides by liquid chromatography-tandem mass spectrometry (LC-MS/MS).

‘Shotgun proteomics’ has also recently emerged as a powerful strategy for the analysis of complex protein mixtures. This approach has been pioneered by mass spectrometric methods such as multi dimensional protein identification technology (MudPIT) (Washburn et al. 2001). Surface-enhanced laser desorption ionization time-of-flight mass spectrometry (SELDI-ToF-MS) has also been employed for proteomics studies (Hutchens and Yip, 1993). This technique has the potential to identify protein biomarkers of disease states but there are still issues regarding reproducibility and further validation is required for biomarker discovery (Baggerly et al. 2004).

Although plasma or serum can be considered as a primary source of biomarkers in animal species (Wait et al. 2002; Miller et al. 2004; Hood et al. 2005) these body fluids present problems of over-representation of a few proteins (e.g. albumin, IgG and transferrin). Immuno-depletion is often used to remove high abundance proteins, however concerns about the use of depletion steps exist due to the limited cross-reactivity of commercial antibodies with proteins from animal species and because low-abundance proteins may be simultaneously removed (Granger et al. 2005). An alternative strategy for reducing the level of abundant proteins from serum samples is the recently developed ‘Protein Equalizer Technology’ (Righetti et al. 2006), which may prove more applicable across species boundaries. An additional challenge in protein biomarker discovery is the enormous complexity of the biological samples. Positional proteomics methods aim to simplify the proteome by isolating either the C- or N-terminal peptides (Gevaert et al. 2003; McDonald et al. 2005). Other approaches to proteome simplification have focused on specific amino acids such as cysteine residues in proteins or peptides (Borisov et al. 2002).

The ability to accurately quantify the concentrations of proteins in a complex mixture is also vital for biomarker applications. Previous proteomic approaches have relied on relative quantitative strategies using methods such as ICAT (Gygi et al. 1999) and iTRAQ (Ross et al. 2004). However, protocols for the absolute quantification of individual proteins (AQUA) (Gerber et al. 2003; Kirkpatrick et al. 2005) and multiple proteins (QconCAT) (Beynon et al. 2005) have recently been developed.

Peptides may be protein derived thus acting as an indicator of protein state, but equally may act in the cell as hormones, growth factors, cytokines or neurotransmitters. Therefore, changes in the concentrations of peptides may be indicative of a diseased state. Peptidomics employs proteomics-based technologies to profile endogenous peptides in tissues and body fluids (Ivanov and Yatskin, 2005; Soloviev and Finch, 2006).

### Use of proteomics in veterinary research

Proteomics is increasingly coming to the forefront of biomarker discovery in veterinary research for a variety of animal diseases.

#### Proteomics for peripartum health prognosis in cows

Cairoli et al. (2006) conducted a study to elucidate differences in protein expression during pregnancy and in the peripartum period in cows with and without postpartum uterine infections. Artificially inseminated Friesian heifers had serum samples taken at monthly intervals over the course of their pregnancies. Serum proteins were separated by gel electrophoresis, and identified by mass spectrometry. Quantitative evaluation of the serum proteome patterns revealed that concentrations of both haptoglobin and orosomucoid/α_1_-acid glycoprotein fluctuated at the time of calving. In cows affected by postpartum endometritis, the concentration of orosomucoid was significantly lower than that of healthy cows. Further investigation and validation of the findings of this study are necessary for biomarkers to be unequivocally identified, but the difference in serum orosomucoid levels holds promise as a prognostic biomarker of postpartum cattle health.

#### Proteomic investigations of porcine respiratory health

In pigs several respiratory tract pathogens can be responsible for respiratory disease and can persist for extended periods of time in convalescent animals. *Actinobacillus pleuropneumoniae* was used as a model to study respiratory infection in swine (Hennig-Pauka et al. 2006). Proteins from bronchoalveolar lavage fluid (BALF) were analyzed by 2-DGE in order to identify changes in proteome expression. Pigs of both sexes were experimentally infected with *A. pleuropneumoniae* and BALF was taken over the course of the infection. Eighty protein spots were found to be differentially expressed in BALF samples taken from individual pigs before and across the 21 day infection period. Twelve proteins were found to be consistently increased at day 21 of infection in all analyses, eight of which were statistically significant. Three of the proteins were identified. These were prophenin-2, PR-39 and calgranulin C. PR-39 was highlighted as a possible biomarker, leading to further analysis of this protein at day 21 of infection. It was found to be consistently elevated and its levels significantly correlated to the lung lesion score. BALF is not the most accessible sample matrix but this study may prove to be a useful starting point for targeted biomarker discovery.

### Metabolomics

Metabolomics monitors alterations in cell function that are perhaps most evident at the level of small molecule metabolism and can provide a coherent view of the response of biological systems to a variety of genetic and environmental influences (German et al. 2005; Oresic et al. 2006). Metabolomics can therefore offer an integrative view of the healthy and the sick animal and provide an additional perspective on the molecular pathogenesis of disease in animals. Importantly, readily accessible metabolites raise the possibility of identifying biomarkers of specific disease states (Serkova and Niemann, 2006). Nonetheless, in metabolomic experiments the effect of analytical and biological influences on metabolite composition of tissues and body fluids needs to be carefully assessed and validated (Bollard et al. 2005; Stanley et al. 2005; Teahan et al. 2006; Wang et al. 2006; Gu et al. 2007).

The wide range of low molecular weight metabolites in complex biological systems demands a variety of analytical platforms for detection, identification and quantification. Suitable techniques must be sensitive, robust and have the capacity to acquire data on metabolite profiles from large populations of samples. Achieving the broadest overview of metabolite composition for biomarker discovery in body fluids such as plasma and urine is challenging and requires an integrated strategy for metabolite analysis and data processing (Dunn et al. 2005).

Ideally, metabolomic analyses should avoid bias for specific molecules and should be able to detect every individual metabolite, a requirement that is only really attained through the use of multiple analytical methods. The techniques most commonly employed in metabolomics analyses are nuclear magnetic resonance (NMR) spectroscopy (Reo, 2002; Viant, 2006), liquid chromatography-mass spectrometry (LC-MS) and gas chromatography-mass spectrometry (GC-MS) (Want et al. 2005; Wilson et al. 2005; Dettmer et al. 2007). Fourier transform-ion cyclotron resonance (FT-ICR) mass spectrometry (Brown et al. 2005) and capillary electrophoresis-mass spectrometry (Soga, 2007) have also aroused interest for the global profiling of low molecular weight metabolites.

Automated methods for both experimental design and maximum metabolite capture have been proposed. (O’Hagan et al. 2005) and initiatives to standardize the reporting of metabolomic analyses have been established (Jenkins et al. 2004; Lindon et al. 2005; Fiehn et al. 2006). However, bioinformatic tools, including mass spectral libraries and deconvolution algorithms, are required to readily identify global populations of low molecular weight metabolites. (Hollywood et al. 2006). Such tools will be critical for biomarker discovery in metabolomics. Due to the large data sets obtained from metabolomics experiments, multivariate data analysis is often employed to provide evidence of metabolite perturbations. These statistical methods provide an efficient, non-biased procedure for interpreting the complex datasets and allow the correlation of metabolic responses in biological systems to be fully investigated (Holmes et al. 2000).

### Metabolomics for biomarker discovery in animals

Whilst still in its infancy, metabolomics strategies have been employed to characterize metabolic changes resulting from altered gene function in plants (Weckwerth et al. 2003; Schauer and Fernie 2006) and to explore microbial metabolism (Kell, 2004) and the mechanisms of drug toxicity (Nicholson et al. 2002; Lindon et al. 2003). Metabolomics is also being used towards diagnostic applications (Brindle et al. 2002; Kenny et al. 2005; Lamers et al. 2005) and to investigate pathophysiological processes in animal models of human diseases (Wang et al. 2004; Griffin, 2006; Major et al. 2006). A number of metabolomics studies have now begun to focus on animal health and disease.

#### Metabolomics and canine hepatology

Metabolomic analyses have been used to characterize metabolic disturbances in canine liver disease (Whitfield et al. 2005). The goal of this study was to employ metabolomics to advance the diagnosis of portovascular anomalies in dogs and provide a means of more accurately assessing the progression of these disease states. In the study, the plasma metabolite profile from three groups of dogs with congenital portovascular anomalies, acquired hepatopathies or unrelated (non-hepatic) disorders was examined using LC-MS. Multivariate data analysis was then used to compare the plasma metabolite profiles of the three groups of dogs. The metabolites which were most significantly increased in dogs with liver disease were identified as the taurine conjugates of the bile acids, cholic and chenodeoxycholic acid whereas 16:0-, 18:2- and 18:0-lysophospha-tidylcholine were decreased in affected animals. In contrast to conventional laboratory measurements, the analysis not only distinguished control and affected cohorts of dogs but also discriminated animals with congenital portovascular anomalies from those with acquired syndromes.

#### Metabolomics approach to monitor the use of anabolic steroids in cattle

The use of beta-agonists, sexual steroids, and corticosteroids as growth-promoting agents in veal calves is forbidden in the European Union (EU) and subjected to restrictions in the U.S. because it may be potentially noxious for both treated animals and the consumer. The presence of these compounds in matrices of biological origin often goes unnoticed because of the use of very low dosages and/or number of molecules of unknown chemical structure. It is therefore necessary to develop methods for the simultaneous screening of large numbers of low molecular weight metabolites. Dumas et al. (2005) investigated the metabolic responses of cattle to anabolic steroid treatment using a metabolomics strategy. Hereford steers were administered a range of steroids and their urine was sampled at various time points. Metabolite profiles were analyzed by NMR spectroscopy and multivariate analysis of the data was performed to reveal metabolites of diagnostic interest. The profile of metabolites involved in nitrogen metabolism (trimethylamine-N-oxide, dimethylamine, hippurate, creatine, creatinine and citrate) was found to be disturbed indicating a coordinated response to anabolic steroids.

### Bioinformatics for biomarker research

Currently data analysis remains a major bottleneck in post-genomic research (Domon and Aebersold, 2006). Biomarker discovery experiments generate large data sets and results are often obtained from the combined endeavors of several laboratories. It is therefore critical that the processing and analysis of complex data sets, incorporating defined standards for data formats, data processing parameters, and data quality assessment, is stream-lined for both ease of use, data exchange and down-stream utility.

Public repositories for genomic and proteomic information such as GenBank and MSDB have contributed greatly to the advances made in post-genomic research (Kersey and Apweiler, 2006). Whilst databases for metabolomic studies are not as well developed (Nobeli and Thornton, 2006), appropriate bioinformatic resources are becoming available (Kopka et al. 2005). Similarly, databases specifically for use in the veterinary sciences have been limited as the genome sequences of most animals have relatively poor structural and functional annotation (McCarthy et al. 2007). The advent of genome projects focused on a number of biologically and economically important animal species are either complete or well advanced and will provide a tremendous tool for those in the field of veterinary research.

## Conclusion

Biomarker discovery has enormous potential for improving animal health and welfare. The rapid advancement of the post-genomic technologies has led to the development of global strategies aimed at relating gene expression to phenotypic outcome in biological systems. These approaches may be used to improve disease diagnosis and prognostic evaluation and as a means of monitoring the efficacy of treatments.

Whilst post-genomic technologies hold great promise for veterinary research, substantial technical challenges remain. Along with the intrinsic problems involved in the global analysis of transcripts, proteins and metabolites, additional issues of feasibility, cost and practicality of using these technologies in a clinical environment should be considered. Of the many biomarkers identified by these approaches few have made their way into routine clinical use. Lack of specificity and sensitivity contribute to the problems faced when bringing a biomarker assay from the laboratory to the clinic and many investigations do not progress to the large-scale studies required for proper biomarker validation.

Post-genomic analyses can be followed by refined experimental approaches, which focus on specific groups of mRNA, proteins or metabolites that are differentially expressed in the initial global profiles. Combining targeted approaches with post-genomic technologies should permit a convergent strategy to integrate biomarker concepts. Such strategies hold great promise for biomarker discovery in animal health and disease.

## Figures and Tables

**Table 1:** Summary of ideal biomarker characteristics.

Characteristics of a Biomarker
Accurate, sensitive and specific for disease state
Biomarker unaffected by unrelated disorders
Reliable quantification of the biomarker from accessible body fluid or tissues
Abundance of biomarker not subject to wide variation in general population
Measurements reproducible and consistent in different circumstances at different times
Biomarker results easy to interpret

**Table 2:** Considerations in biomarker validation.

Sources of Variability	
**Biological**	**Analytical**
Species and breed of animal	Type of specimen
Sex	Type of sample
Age	Sample collection
Neuter status	Temperature of storage
Hormonal status and pregnancy	Duration of storage
Diurnal variation	Type of assay
Diet	Sensitivity of assay
Animal handling and environment	Specificity of assay

**Table 3:** Example applications of post-genomics technologies to animal health and disease.

Animal	Application	Body Fluid or Tissue	Reference
**Transcriptomics**			
Chicken	Marek’s disease	White blood cells	Liu et al. 2001
Cow	Parasite tolerance	White blood cells	Berthier et al. 2003
Cow	Mastitis	White blood cells	Park et al. 2004
Cow	Johne’s disease	White blood cells	Skovgaard et al. 2006
Dog	Osteoarthritis	Cartilage	Burton-Wurster et al. 2005
Dog	Pancreatic acinar atrophy	Pancreas	Clark et al. 2005
Dog	Dilated cardiomyopathy	Heart	Oyama and Chittur, 2005
Dog	Cancer	Brain tumor	Thomson et al. 2005
Dog	Renal disease	Kidney	Greer et al. 2006
Horse	Osteoarthritis	Cartilage	Smith et al. 2006
Pig	Pathogen detection	Porcine pathogens	Liu et al. 2006
Sheep	Disease resistance	Duodenum	Keane et al. 2006

**Proteomics**			
Cow	Follicular cysts	Follicular fluid	Maniwa et al. 2005
Cow	Peripartum health diagnosis	Serum	Cairoli et al. 2006
Fish	Cancer	Plasma	Ward et al. 2006
Horse	Infection biology	Serum	Roncada et al. 2005
Horse	Connective tissue injury	Tendon	Sodersten et al. 2006
Pig	Respiratory infection	Bronchoalveolar lavage fluid	Hennig-Pauka et al. 2006
Sheep	Copper toxicosis	Liver	Simpson et al. 2004

**Metabolomics**			
Cow	Monitoring steroid use	Urine	Dumas et al. 2005
Dog	Liver disease	Plasma	Whitfield et al. 2005
Fish	Cancer	Liver	Stentiford et al. 2005
